# Examining the etiological factors resulting in retinal detachment following prophylactic vitrectomy in the context of acute retinal necrosis syndrome

**DOI:** 10.1186/s12886-024-03518-2

**Published:** 2024-06-13

**Authors:** Yu-hong Nie, Yu Zhang, Zhen Chen, Yi-qiao Xing

**Affiliations:** Department of Ophthalmology, Renmin Hospital of Wuhan University, No. 99 of Zhangzhidong Road, Wuchang District, Wuhan, 430060 China

**Keywords:** Acute retinal necrosis syndrome, Prophylactic pars Plana Vitrectomy, Proliferative vitreoretinopathy, Retinal detachment, Retinal necrosis

## Abstract

**Objective:**

The aim of this study is to elucidate the factors contributing to the occurrence of retinal detachment (RD) following prophylactic vitrectomy in cases of acute retinal necrosis (ARN) syndrome.

**Methods:**

A retrospective examination was undertaken, encompassing the medical records of patients diagnosed with ARN who underwent prophylactic vitreous intervention at the Ophthalmology Department of Wuhan University Renmin Hospital East Campus between October 2019 and September 2023. Subsequently, patients who manifested RD in the postoperative period were identified, and a comprehensive analysis was conducted to ascertain the factors underlying the occurrence of RD post-surgery.

**Results:**

This study comprised 14 cases (involving 14 eyes) of patients diagnosed with ARN who underwent prophylactic vitreous intervention. The findings revealed that 4 patients experienced postoperative RD, resulting in an incidence rate of 28.57%. Notably, among these cases, 3 cases of RD manifested in the presence of silicone oil, while 1 case occurred subsequent to the removal of silicone oil. All 4 cases of RD exhibited varied degrees of proliferative vitreoretinopathy. Following the occurrence of RD, all patients underwent a secondary vitreous intervention coupled with silicone oil tamponade, leading to successful reattachment of the retina. However, despite these interventions, there was no significant enhancement observed in postoperative visual outcomes when compared to preoperative levels.

**Conclusion:**

RD following prophylactic vitrectomy in cases of ARN is not an infrequent occurrence and is primarily linked to the postoperative onset of proliferative vitreoretinopathy.

## Introduction

Acute retinal necrosis (ARN) syndrome, caused by infections such as herpes simplex virus (HSV), varicella-zoster virus (VZV), cytomegalovirus (CMV), and Epstein-Barr virus (EBV), constitutes a severe and vision-threatening blinding eye disease with an unfavorable prognosis [1, 2]. It primarily manifests as severe uveitis, necrotizing retinitis, occlusive arteritis, and eventually progresses to retinal detachment (RD). Contemporary therapeutic approaches for ARN primarily involve systemic administration of antiviral agents, supplemented by glucocorticoids and antithrombotic medications, with consideration given to vitrectomy in advanced cases [3]. Prophylactic vitrectomy, refers to the performance of a pars plana vitrectomy (PPV) prior to the onset of RD in ARN, which has emerged as a therapeutic strategy [4]. Recent studies have posited that prophylactic PPV combined with systemic antiviral treatment may reduce the incidence of RD in patients diagnosed with ARN [5–7]. A meta-analysis indicated that the combined application of prophylactic PPV and systemic antiviral treatment reduces the incidence of RD in patients with ARN to 18% [8]. Despite the demonstrated efficacy of this combined intervention in diminishing RD rates, a substantial proportion of patients with ARN still experience RD following prophylactic PPV, thereby exerting a profound impact on the recovery of visual function. Consequently, a retrospective examination of medical records pertaining to patients with ARN who developed RD subsequent to prophylactic PPV within our institution was undertaken. The objective of our study was to explore the causes of retinal detachment after prophylactic vitrectomy for ARN, in the hope that these factors could be avoided in the subsequent treatment of ARN, or a more appropriate treatment could be selected according to the patient’s condition.

## Materials and methods

### General information

This study included 14 patients (comprising 14 eyes) who underwent prophylactic PPV for ARN at the Ophthalmology Department of Wuhan University Renmin Hospital East Campus during the period from October 2019 to September 2023. The decision to implement prophylactic PPV in cases of ARN was primarily contingent upon the advancement of necrotic lesions. Surgical indications were predominantly guided by established criteria cited in literature [6], including: (1) involvement of retinal necrosis lesions in the posterior pole or proximity to the posterior pole; (2) rapid progression of retinal necrosis lesions despite antiviral drug treatment; (3) manifestation of severe vitreous opacification. Exclusion criteria encompassed patients diagnosed with ARN who had already developed RD.

### ARN diagnostic criteria

The diagnosis of ARN adhered primarily to the criteria established by the American Uveitis Society in 1994 [9]. These criteria comprised the identification of: (1) one or more well-demarcated lesions on the peripheral retina; (2) rapid progression of the disease in the absence of antiviral treatment; (3) circumferential progression of the disease; (4) indications of occlusive retinal vasculitis affecting the arteries; and (5) a pronounced inflammatory reaction evident in both the anterior chamber and vitreous. The classification of the extent of retinal necrosis was conducted in accordance with literature guidelines [10]: Zone 1 denotes the region within 3000 μm (2-disc diameters) of the macular fovea center or 1500 μm from the optic disc; Zone 2 extends anteriorly from Zone 1 to the equator; Zone 3 encompasses the region from Zone 2 anteriorly to the ora serrata.

### Methods

All patients received a standardized preoperative regimen of full antiviral drug treatment, administered intravenously with acyclovir injection at a dosage of 10 mg/kg thrice daily. Following 7 to 10 days of continuous intravenous therapy, the treatment modality transitioned to oral acyclovir, administered at 800 mg five times daily, spanning a duration of 6 to 14 weeks. Some patients received glucocorticoids, and in specific cases, ocular fluids (aqueous humor or vitreous) were extracted for viral nucleic acid testing. A subgroup of patients additionally underwent intravitreal antiviral medication via ganciclovir injection at a dosage of 400 µg/0.1 ml. Topical anti-inflammatory treatment, involving tobramycin and dexamethasone eye drops, along with pupil dilation through a compounded tropicamide solution, was also administered. All patients underwent comprehensive pre- and postoperative assessments, encompassing evaluations of best-corrected visual acuity (BCVA), intraocular pressure, slit-lamp microscopy, fundus pre-set lens examination, and fundus photography. Visual acuity values were converted to LogMar for representation.

The surgical interventions were uniformly conducted by a single surgeon possessing extensive expertise in vitreous retinal surgery. Before the surgery, the operative eye underwent dilation with a compounded tropicamide solution, followed by surface and retrobulbar anesthesia. A 23G trocar was utilized for puncture at the standard vitrectomy three-port sites (3–4 mm posterior to the limbus). In cases where vitreous detachment was not naturally occurring, manual aspiration was performed using a vitreous cutter. The turbid vitreous in central and peripheral regions was thoroughly removed, adopting a high cutting speed and low negative pressure mode for vitrectomy in front of necrotic lesions. Laser photocoagulation was applied in 3 to 5 rows along the borders of retinal necrosis lesions, followed by a fluid-air exchange and the infusion of silicone oil into the vitreous cavity. Postoperative management encompassed continued antiviral medications, glucocorticoids, and symptomatic treatment. Patients were advised to maintain a prone position for at least one week. Subsequent follow-up evaluations were conducted for a minimum period of six months postoperatively.

### Statistical analysis

The statistical analysis of the collected data was carried out using SPSS17.0 software. Quantitative data are expressed as mean ± standard deviation. The comparison of BCVA values before and after surgery was performed using paired *t*-tests, with *P* < 0.05 considered statistically significant.

## Results

### Clinical characteristics of enrolled patients

Table 1 presents the demographic and clinical characteristics of the cohort consisting of 14 patients (14 eyes) who underwent PPV for ARN. Among the participants, 8 were male and 6 were female, with ages ranging from 24 to 65 years and an average age of 49.00 ± 13.61 years. The duration from the onset of symptoms to diagnosis varied from 7 to 30 days, with an average of 14.57 days. All patients exhibited retinal necrosis lesions involving Zone 2, with 4 patients (28.57%) having retinal involvement in all four quadrants, 9 patients (64.28%) in three quadrants, and 1 patient (7.15%) in two quadrants. Optic nerve involvement was observed in 2 patients (7.15%, patients 3 and 10). Two patient (patient 1 and 13) had undergone prophylactic laser photocoagulation previously. PCR testing of ocular fluids was conducted in 3 patients (patients 1, 9, and 10), revealing VZV infection in all cases, with viral DNA copy numbers of 4.53 × 10^6^ /ml, 1.06 × 10^7^ /ml, and 5.84 × 10^6^ /ml, respectively. Intravitreal ganciclovir injections were administered to 3 patients (16.67%, patients 1, 2 and 13). Prophylactic PPV was undertaken for various indications: 5 patients (patients 5, 8, 10, 11, and 12) due to retinal necrosis lesions approaching the posterior pole; 3 patients (patients 2, 9 and 13) due to rapid progression of retinal necrosis lesions despite antiviral drug treatment; and 6 patients (patients 1, 3, 4, 6, 7 and 14) due to severe vitreous opacification. The time from diagnosis to PPV for the 14 patients diagnosed with ARN ranged from 1 to 20 days, with an average of 3.21 days. All patients underwent prophylactic PPV combined with silicone oil tamponade. Follow-up periods ranged from 6 to 35 months, with an average of 23.81 months.

**Table 1:** Clinical Features of patients with ARN who underwent prophylactic vitreous cutting

Patient	Gender	Age	Duration from onset to first visit (days)	Extent of retinal necrosis area	Retinal necrosis quadrants	Preoperative visual acuity	Duration from diagnosis to PPV	RD occurrence postoperatively	Final follow-up visual acuity
1	Male	61	15	Zone 2	4	0.08	20	No	0.02
2	Male	43	15	Zone 2	3	HM	4	No	0.05
3	Male	54	10	Zone 2	4	HM	1	Yes	FC
4	Male	24	7	Zone 2	3	0.1	2	No	0.2
5	Female	45	20	Zone 2	2	0.3	1	No	0.4
6	Female	60	7	Zone 2	3	0.12	1	No	HM
7	Female	64	20	Zone 2	3	0.02	1	No	0.6
8	Female	48	30	Zone 2	4	FC	2	Yes	FC
9	Female	56	14	Zone 2	3	0.2	3	Yes	HM
10	Male	27	7	Zone 2	3	FC	1	Yes	FC
11	Male	30	10	Zone 2	3	0.1	2	No	0.3
12	Male	56	20	Zone 2	3	HM	1	No	FC
13	Femal	65	14	Zone 2	3	0.1	4	No	0.12
14	Male	53	15	Zone 2	4	0.05	2	No	0.05

### Occurrence of RD following prophylactic PPV in patients with ARN

**Fig. 1:**
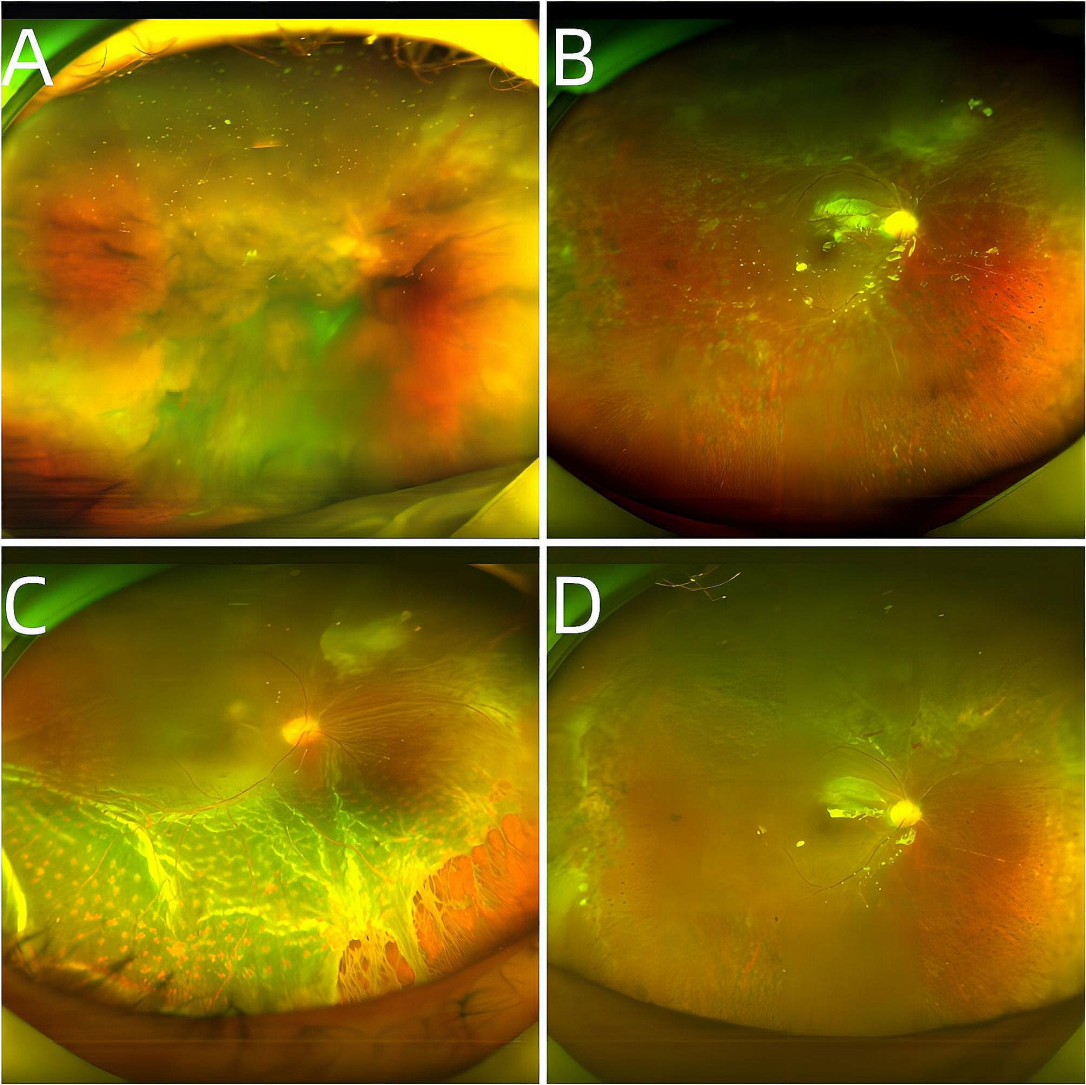
Fundus photos of a patient before and after surgery. (**A**) Fundus photograph of 30 days after the onset of the disease, showing obvious vitreous opacity; (**B**) Fundus photographs of 3 months after the first vitrectomy and filling with silicone oil showed retinal necrosis and vascular occlusion; (**C**) One month after vitreous silicone oil removal, fundus photography showed retinal proliferation and traction to form holes and massive retinal detachment; (**D**) Fundus photographs taken 1 week after the second vitrectomy showed that the retina was flat and the peripheral retina was still proliferating

Table 2 reveals the incidence of RD following PPV in patients with ARN. Among the participants, 3 patients (patients 3, 8, and 9) developed RD while in a silicone oil-filled state, while 1 patient (patient 10) developed RD one week after the removal of silicone oil. The overall incidence rate of RD in this group was 28.57%. The time to RD post-PPV varied for patients 3, 8, 9, and 10, being 180, 115, 53, and 137 days post-ARN diagnosis, respectively. All four patients with RD manifested varying degrees of proliferative vitreoretinopathy (PVR) post-PPV, with PVR onset occurring approximately 6 weeks after the surgical intervention and progressively worsening over time. Notably, 1 patient (patient 10) exhibited a gradual worsening of retinal artery occlusion in the postoperative period, leading to hypotony (ranging between 2 and 5 mmHg), ultimately resulting in RD one week after silicone oil remova (Fig. 1). Subsequent to the occurrence of RD, all 4 patients underwent a second PPV, encompassing procedures such as membrane peeling, retinotomy, laser photocoagulation, and silicone oil tamponade. Postoperatively, retinal reattachment was achieved in 3 patients (patients 3, 9, and 10). However, 2 of these patients (patients 8 and 9) experienced recurrent PVR.

**Table 2:** Situation of postoperative RD in patients with ARN receiving prophylactic PPV

Patient	Duration to RD postoperatively (days)	State at RD occurrence	Concurrent PVR	Retinal status after second surgery
3	180	Silicone oil-filled state	Yes	Reattached
8	115	Silicone oil-filled state	Yes	Recurrent PVR
9	53	Silicone oil-filled state	Yes	Reattached, recurrent PVR
10	137	After silicone oil removal	Yes	Reattached

### Preoperative and postoperative BCVA in patients with ARN

Six eyes (42.86%) of patients with ARN had a BCVA below 0.05 prior to surgery, and 8 eyes (57.14%) had a BCVA between 0.05 and 0.3. Seven eyes (50%) had a BCVA below 0.05 at the conclusion of the postoperative follow-up period, 5 eyes (35.71%) had a BCVA between 0.05 and 0.3, and two eyes (14.29%) had a BCVA above 0.3. Following surgery, BCVA improved in 8 patients, representing a 57.14% increase in visual acuity. In 3 cases (21.43%), the BCVA remained unaltered, however in 3 patients (21.43%), there was a visual decrease. Preoperatively, the mean BCVA was 1.59 ± 0.88logMar, while postoperatively, it was 1.50 ± 0.89logMar. There was no significant difference in BCVA between before and after surgery (*t* = 0.251, *P* = 0.804 > 0.05).

### Changes in BCVA before and after surgery in patients with ARN who developed RD

Among the four patients with ARN who experienced RD subsequent to prophylactic PPV, no significant improvement in visual acuity was observed following the second surgical intervention. Specifically, in one patient, visual acuity decreased from 0.2 preoperatively to perceiving hand motion. Two patients exhibited no change in visual acuity, while one patient demonstrated an improvement from perceiving hand motion to counting fingers.

## Discussion

ARN is a highly destructive ocular disease characterized by a challenging prognosis, underscoring the critical importance of timely diagnosis and intervention to enhance clinical outcomes. Given the rarity of ARN, the absence of large-scale randomized controlled trials has resulted in a lack of established definitive treatment guidelines. Presently, the primary objectives in ARN treatment focus on averting visual deterioration and mitigating the risk of RD.

The likelihood of RD in ARN varies widely, ranging from 20 to 73%, and the elevated incidence of RD significantly contributes to the unfavorable visual prognosis associated with ARN [11]. Recognizing that RD often manifests subsequent to the acute phase of ARN, there is a growing body of research advocating for early prophylactic PPV to diminish RD incidence and enhance overall prognosis. Prophylactic PPV serves to clear inflammatory mediators, alleviate inflammatory responses, diminish vitreous traction on the retina, and facilitate extensive photocoagulation behind the necrotic retina during surgery, and employs long-acting tamponades to prevent subsequent RD [11]. The efficacy of prophylactic PPV in treating ARN remains a topic of debate.Huang et al. [12] conducted a comparative analysis between patients diagnosed with ARN who underwent early PPV and those who did not, revealing a lower RD incidence of 25% (3/12) in the early PPV group compared to 59% (10/17) in the non-early PPV group, indicating a potential benefit of early PPV within 30 days in preventing RD. In contrast, Risseeuw et al. [4] reported a lower but still notable incidence of RD following prophylactic PPV in ARN, with a rate of 14.3% (1/7). Despite these positive findings, conflicting results and uncertainties persist in literature, necessitating further research to delineate the efficacy of prophylactic PPV in ARN [4, 11, 13, 14]. Systematic reviews and meta-analyses suggest that while prophylactic PPV may reduce the risk of RD, it could concomitantly increase the risk of PVR [8, 15]. Notably, the probability of RD occurrence after PPV varies widely, spanning from 0 to 94% [8]. In the present study, the observed incidence of RD following prophylactic PPV in ARN was 28.57%, with three cases occurring in a silicone oil-filled state and one case after silicone oil removal. This highlights that RD occurrence after prophylactic PPV is not uncommon. Consequently, there is a compelling need to investigate the causative factors and variations contributing to RD after prophylactic PPV in ARN, emphasizing the significance of further research in this domain.

Currently, there exists a limited body of literature elucidating the etiological factors contributing to RD subsequent to prophylactic PPV in ARN. Based on the findings of this retrospective study and relevant scholarly works, we postulate that the manifestation of RD is correlated with the following factors:

(1) Temporal alignment with the PPV procedure: If retinal proliferation is underway at the time of PPV, RD may manifest postoperatively notwithstanding the surgical intervention.

(2) Correlation with incomplete posterior vitreous detachment during PPV and insufficient vitreous removal at the base: Ishida et al. [6] reported that induced posterior vitreous detachment during prophylactic PPV in ARN may result in iatrogenic breaks. To avoid the risk of such breaks, incomplete posterior vitreous detachment may transpire, given the peripheral location of inflammatory ARN lesions, necessitating meticulous peripheral vitreous removal. However, due to tissue fragility, the susceptibility to iatrogenic breaks during PPV is heightened. These factors collectively predispose patients to PVR, subsequently culminating in RD.

(3) Association with the extent of the area affected by preoperative retinal necrosis lesions: the study conducted by Ishida et al. [6] proposes that the initial extent of necrotizing retinitis can serve as a predictive parameter for RD development following prophylactic PPV. In patients having ARN with necrotic lesions encompassing Zone 1, RD may persist despite prophylactic PPV; conversely, prophylactic PPV proves efficacious in preventing RD in cases where ARN lesions are confined to Zone 2. Moreover, ARN lesions limited to Zone 3 can be successfully treated with antiviral medication alone. Using multivariate analysis, Risseeuw et al. [4] substantiated the correlation between RD occurrence and the extent of retinal area affected by necrosis. The risk of RD is diminished when retinitis is confined to Zone 3, but escalated when Zones 1 and 2 are involved. Zhao et al. [8] posited that the likelihood of RD is contingent upon the number of retinal quadrants involved, the extent of the affected area, the viral infection type, and immune status. Cumulatively, these investigations signify an elevated risk of RD in cases where ARN necrotic lesions involve Zones 1 and 2, with RD persisting even in the presence of prophylactic PPV. In our study group, all patients exhibited retinal necrosis involving Zone 2, with 28.57% displaying involvement in all four quadrants, 64.28% in three quadrants, and 7.15% in two quadrants.

(4) High viral load in VZV infections: The assessment of viral diagnostic testing on ocular fluids in ARN serves as a valuable tool for guiding subsequent treatment strategies and enhancing prognostic understanding. Noteworthy findings indicate that vision impairment in patients diagnosed with ARN attributed to VZV is more severe compared to that resulting from HSV [16]. Additionally, a conjecture exists that a viral DNA copy number equal to or exceeding ≥ 5.0 × 10^6^/ml may signify a more pronounced uveitis reaction, diminished visual acuity, and an elevated incidence of RD. In this study, two out of four patients having RD tested positive for VZV, exhibiting viral DNA copy numbers surpassing ≥ 5.0 × 10^6^/ml, thus suggesting an increased vulnerability to RD.

(5) Occlusive vasculitis inducing retinal ischemia and thinning: the progression of occlusive vasculitis in ARN exacerbates retinal ischemia, resulting in retinal thinning, atrophy, the formation of retinal breaks, and subsequently, RD [16]. In this study, post-PPV, patient 10 exhibited a pronounced occlusion of retinal arteries, leading to retinal thinning and atrophy, culminating in RD subsequent to the removal of silicone oil.

(6) Postoperative onset of PVR: Liu et al. [14] observed that all patients with ARN, irrespective of their affiliation with either the regular antiviral treatment group or those undergoing prophylactic PPV, eventually developed PVR, thereby establishing a correlation between post-PPV RD and PVR. Furthermore, Zhao et al. reported a 32% incidence of PVR development in patients with ARN following prophylactic PPV [8]. In the current study, all four patients with ARN exhibited varying degrees of PVR post-PPV, resulting in diverse extents of retinal traction and subsequent RD. Consequently, PVR emerges as a primary causative factor for postoperative RD, underscoring the paramount significance of PVR prevention subsequent to ARN surgery. The diverse outcomes observed following prophylactic PPV in patients with ARN may be attributed to factors such as the viral type infecting the patient, viral DNA load, the immune status of the patient, the inflammatory response degree, and the extent of retinal necrosis.

In this study, PPV in patients with ARN resulted in a significant enhancement of visual acuity in certain patients, whereas others encountered a noteworthy decline in visual function. However, statistical analysis failed to reveal a substantial difference, indicating that prophylactic PPV does not yield a consistent improvement in the visual prognosis of patients with ARN. This inconsistency may be attributed to potential selection bias within the cases, leading to a restricted range of vision recovery. Additionally, optic disc pathology, macular edema, the presence of an epiretinal membrane, and postoperative hypotony were identified as factors influencing visual prognosis.

## Conclusion

In summary, the administration of prophylactic PPV, when coupled with systemic and intravitreal antiviral treatment, demonstrated a capacity to reduce the incidence of RD in patients with ARN. Notably, in cases marked by moderate to severe necrosis and substantial vitreous opacification, prophylactic vitreous removal surgery emerges as a viable option. The onset of postoperative RD may be linked to several factors, including the timing of the surgery, incomplete removal of inflammatory vitreous, the extent of retinal necrosis, the nature of the infecting virus, occlusive vasculitis, and the development of PVR.

## Data Availability

The datasets generated and analysed during the current study are not publicly available but are available from the corresponding author （Yi-qiao Xing）on reasonable request.

## Abbreviations

ARN: Acute retinal necrosis syndrome
HSV: Herpes simplex virus
VZV: Varicella-zoster virus
CMV: Cytomegalovirus
RD: Retinal detachment
PPV: Pars plana vitrectomy
BVCA: Best corrected visual acuity
PVR: Proliferative vitreoretinopathy
